# Tumor-associated antigen Prame targets tumor suppressor p14/ARF for degradation as the  receptor protein of CRL2^Prame^ complex

**DOI:** 10.1038/s41418-020-00724-5

**Published:** 2021-01-27

**Authors:** Wenjuan Zhang, Lihui Li, Lili Cai, Yupei Liang, Junfeng Xu, Yue Liu, Lisha Zhou, Chen Ding, Yanmei Zhang, Hu Zhao, Jun Qin, Zhimin Shao, Wenyi Wei, Lijun Jia

**Affiliations:** 1 Cancer Institute, Longhua Hospital, Shanghai University of Traditional Chinese Medicine, Shanghai, 200032 China; 2 Department of Breast Surgery, Key Laboratory of Breast Cancer in Shanghai, Fudan University Shanghai Cancer Center, Shanghai, 200032 China; 3 Cancer Institute, Fudan University Shanghai Cancer Center, Shanghai, 200032 China; 4 Department of Clinical Laboratory, Huadong Hospital, Fudan University, Shanghai, 200040 China; 5 Shanghai Key Laboratory of Clinical Geriatric Medicine, Fudan University, Shanghai, 200040 China; 6 Research Center on Aging and Medicine, Fudan University, Shanghai, 2000402 China; 7 State Key Laboratory of Proteomics, Beijing Proteome Research Center, Beijing Institute of Radiation Medicine, Beijing, 102206 China; 8 National Center for Protein Sciences, The PHOENIX Center, Beijing, 102206 China; 9 Department of Pathology, Beth Israel Deaconess Medical Center, Harvard Medical School, Boston, 02115 MA USA

**Keywords:** Tumour-suppressor proteins, Neddylation, Ubiquitin ligases, Protein-protein interaction networks

## Abstract

Protein Preferentially Expressed Antigen in Melanoma (Prame), a tumor-associated antigen, has been found to frequently overexpress in various cancers, which indicates advanced cancer stages and poor clinical prognosis. Moreover, previous reports noted that Prame functions as a substrate recognizing receptor protein of Cullin RING E3 ligases (CRLs) to mediate potential substrates degradation through Ubiquitin Proteasome System (UPS). However, none of the Prame specific substrate has been identified so far. In this study, proteomic analysis of RBX1-interacting proteins revealed p14/ARF, a well-known tumor suppressor, as a novel ubiquitin target of RBX1. Subsequently, immunoprecipitation and in vivo ubiquitination assay determined Cullin2-RBX1-Transcription Elongation Factor B Subunit 2 (EloB) assembled CRL2 E3 ligase complex to regulate the ubiquitination and subsequent degradation of p14/ARF. Finally, through siRNA screening, Prame was identified as the specific receptor protein responsible for recognizing p14/ARF to be degraded. Additionally, via bioinformatics analysis of TCGA database and clinical samples, Prame was determined to overexpress in tumor tissues vs. paired adjacent tissues and associated with poor prognosis of cancer patients. As such, downregulation of Prame expression significantly restrained cancer cell growth by inducing G2/M phase cell cycle arrest, which could be rescued by simultaneously knocking down of p14/ARF. Altogether, targeting overexpressed Prame in cancer cells inactivated RBX1-Cullin2-EloB-Prame E3 ligase (CRL2^Prame^) and halted p14/ARF degradation to restrain tumor growth by inducing G2/M phase cell cycle arrest.

## Introduction

CRL is the largest multiunit E3 ubiquitin ligase family that regulates diverse biological processes through mediating the ubiquitination and degradation of a variety of substrates including signal transducers, cell cycle regulators, transcription factors, tumor suppressors and oncoproteins [1–3]. The CRL complex contains one of  Cullin proteins (Cullin 1, 2, 3, 4A/4B, 5 and 7) that functions largely as molecular scaffold, an adaptor protein alone or together with a substrate specific receptor protein at the N-terminus and a RING protein (RBX1/Roc1 or SAG/Roc2) at the C-terminus [1–3]. Activation of CRL requires the expression of those essential components consisting CRL complex as well as neddylation modification on Cullin protein [4–6]. Protein neddylation is a three-step enzymatic cascade, which conjugates neural precursor cell expressed developmentally downregulated 8 (NEDD8), a ubiquitin-like molecule, to targeted proteins and thus modulates multiple biological processes [7–11]. Once NEDD8 is attached to the C-terminus lysine residue of Cullin [4, 6], the structure of CRL complex would change from a closed conformation to an open one via conformational rearrangement, which subsequently leads to CRL activation [5, 12, 13].

Prame is a tumor-associated antigen that was first identified in metastatic cutaneous melanoma and is subsequently found to frequently overexpress in various cancers, which also associates with advanced stages and poor clinical outcomes [14–16]. On the contrast, normal healthy tissues are not known to express Prame except for testis, ovary, placenta, adrenals and endometrium [17]. Because of its expression profile, targeting Prame for cancer treatment and exploring it as a potential biomarker for diagnosis or prognosis arouse great clinical interest in recent years [15, 18, 19]. At the N-terminus of Prame, there is a BC box or Cul-2 box mediating the interaction with Cullin2 [19, 20]. The C-terminus of Prame is the consensus LXXLL-binding domain mediating the interaction with potential substrates [19, 20]. However, substrate of CRL2^Prame^ E3 ligase complex has not been reported so far [21].

As the core structure of CRL, RBX1 is a high evolutionarily conserved RING-H2 finger domain-containing protein, which interacts with all Cullin family members except Cullin 5, and is required for CRL E3 ligase activity [22, 23]. In addition to function as the essential component of CRL, RBX1 also serves as a NEDD8 E3 ligase to promote Cullin (1, 2/5, 3, 4A/4B and 7) neddylation [24, 25]. Previous studies reported that RBX1 is overexpressed in several human cancers and associates with poor prognosis of cancer patients [26–28]. In contrast, downregulation of RBX1 expression triggers multiple death and growth arrest pathways to effectively suppress the malignant phenotypes of cancer cells [26–28]. These findings highlight RBX1 as an oncogenic factor during tumorigenesis and an attractive target for cancer therapy.

Alternate Reading Frame (ARF), known as p14/ARF in human and p19/ARF in mouse, is encoded by Ink4a/ARF (CDKN2A) locus that also encodes the p16/INK4A tumor suppressor [29]. ARF null-mice develop spontaneous tumors at an early age, demonstrating its tumor suppressive functions [30–32]. One of the most well-defined functions of ARF is to suppress aberrant cell growth in response to oncogenic insults in part through activating the p53 tumor suppressive pathway [33–35]. By blocking ubiquitin ligases MDM2 (MDM2 proto-oncogene) and ARF-BP1/Mule (ARF-binding protein1/Mcl1-ubiquitin ligase E3) mediated p53 degradation, ARF stabilizes and stimulates p53 activity [36, 37]. ARF also has the ability to restrain cell growth independently of p53. To this end, p53-independent function of ARF is thought to attenuate ribosomal RNA transcription and processing in part by binding to NPM1 (nucleophosmin1) [38, 39]. Moreover, ARF interacts with and antagonizes the transcriptional function of Myc and E2F1 independently of p53 [37]. ARF also prevents angiogenesis by limiting the translation of existing VEGFA mRNAs [40]. As an important tumor suppressor, the expression of ARF is tightly controlled at transcriptional and post-translational levels [41, 42]. Previous study reported that β-TrCP2, an F-box protein as well as a substrate receptor of SKP1–Cullin1–F-box protein (SCF, a subfamily of CRL E3 ligases) complex, regulates the degradation of p19/ARF, but not p14/ARF [43]. Therefore, it is unclear whether CRL E3 ligase regulates p14/ARF degradation.

We reported here that, by utilizing a non-biased proteomics approach, p14/ARF is identified as a novel ubiquitin substrate of CRL2^Prame^ in human cancers. Moreover, substrate recognizing receptor protein Prame is determined to be overexpressed in human cancers and negatively correlated with patients’ prognosis. Targeting overexpressed Prame in human cancer cell lines blocks p14/ARF degradation and induces G2/M phase cell cycle arrest, thus inhibits cancer cell proliferation. Our study reveals a previously unknown regulatory mechanism of p14/ARF in human cancer and validates CRL2^Prame^ E3 ligase complex as a promising anti-cancer target.

## Results

### P14/ARF is identified as a novel RBX1-interacting protein

**Fig. 1:**
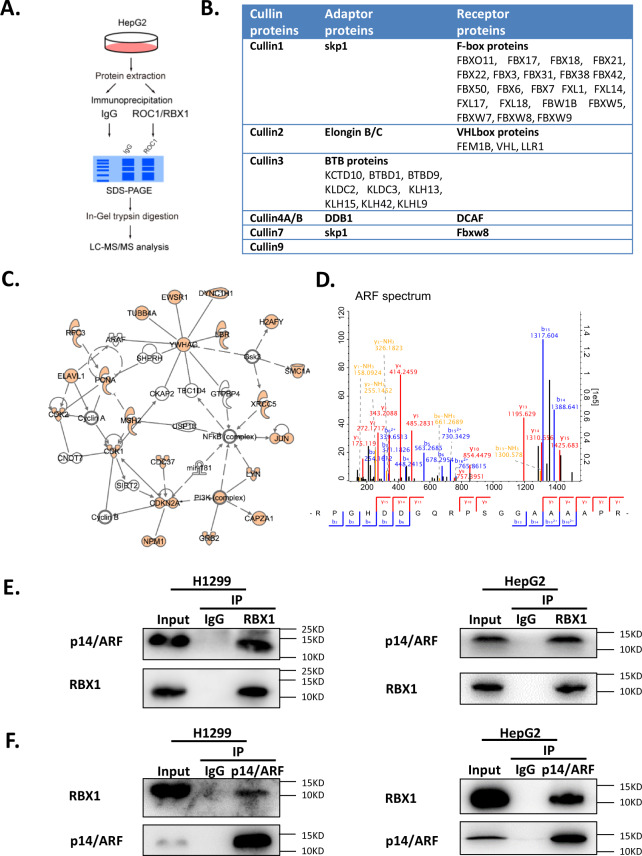
P14/ARF is identified as a novel RBX1-interacting protein. **A** A schematic view of mass spectrometry analysis of RBX1 interacting proteins. **B** Already known RBX1 interacted proteins (Cullin proteins, adaptor proteins, and receptor proteins) were detected via mass spectrometry analysis. **C** Network analysis of identified endogenous RBX1-interacting proteins. **D** Representative tandem MS peptide spectrum of p14/ARF. **E** RBX1 interacts with p14/ARF at endogenous levels. H1299 and HepG2 cells were pretreated with MG-132 for 2 h (hours). Cells were harvested and subjected to immunoprecipitation with anti-RBX1 Ab and immunoblotting with anti-p14/ARF Ab. **F** Endogenous p14/ARF interacts with RBX1 in H1299 and HepG2 cell lines. H1299 and HepG2 cells were pretreated with MG-132 for 2 h. Cells were harvested and subjected to immunoprecipitation with anti-p14/ARF Ab and immunoblotting with anti-RBX1 Ab.

Given that RBX1, the core subunit of CRL E3 Ligases, has been well identified as an oncogenic factor, we performed proteomic analysis of RBX1-interacting proteins to reveal potential mechanisms underlying RBX1-regulating biological effects. Immunoprecipitation with RBX1 Ab followed by mass spectrometry was performed to identify those proteins specifically interacting with RBX1 in cells (Fig. 1A). As shown in Fig. 1B, those well-known RBX1-interacting proteins such as Cullins (Cullin 1–3, 4A, 4B, 7, 9), corresponding adaptor proteins (Skp1, EloB/C, DDB1, etc.) and target recognizing subunits (substrate receptor proteins, like F-box, VHL box, BTB proteins and DCAF) were well detected, making this mass spectrometry analysis a quite credible one. Besides those well-known RBX1 interacting proteins, a large number of potential RBX1-interacting proteins were also detected (Fig. 1C). Among them, the *CDKN2A* encoding tumor suppressor p14/ARF was identified (Fig. 1C, D). Immunoprecipitation assay with anti-RBX1 Ab and immunoblotting with anti-p14/ARF Ab validated the interaction between RBX1 and p14/ARF at endogenous level in cells (Fig. 1E), which was further confirmed by reverse immunoprecipitation with anti-p14/ARF Ab and immunoblotting with anti-RBX1 Ab (Fig. 1F). Collectively these findings indicated that p14/ARF was a novel RBX1-interacting protein in cells.

### RBX1 regulates the degradation of p14/ARF through ubiquitin proteasome system

**Fig. 2:**
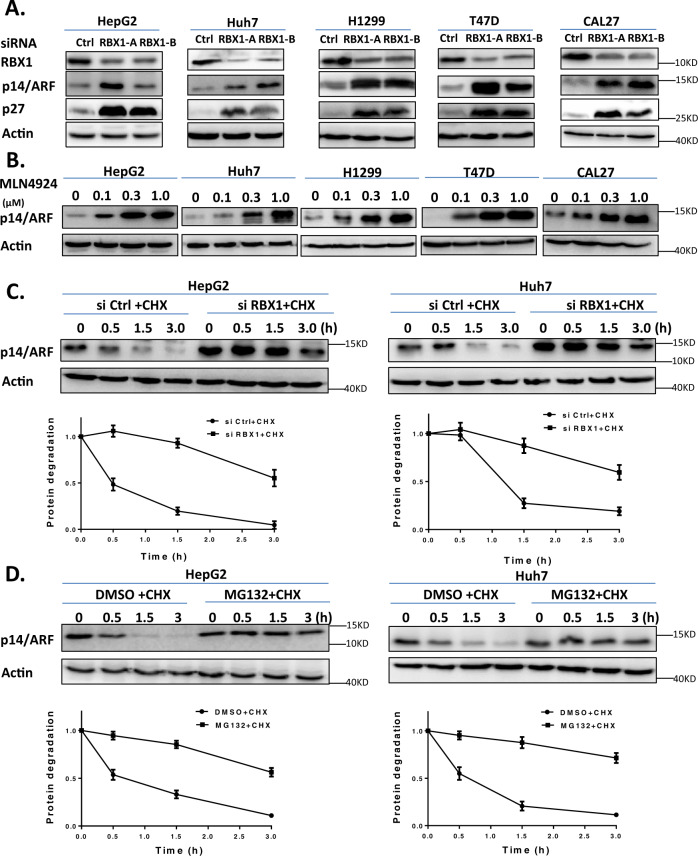
RBX1 mediates degradation of p14/ARF. **A** Downregulation of *RBX1* induces p14/ARF accumulation in HepG2, Huh7, H1299, T47D and CAL27 cells lines. Cells were transfected with control (Ctrl) or RBX1 siRNA for 96 hours (h) and subjected for immunoblotting analysis using antibodies against RBX1 and p14/ARF with Actin as a loading control. **B** MLN4924 treatment induces p14/ARF accumulation in a dose-dependent manner. HepG2, Huh7, H1299, T47D and CAL27 cells were treated with MLN4924 at increasing concentrations (0.1, 0.3, 1.0 μmol/L) versus DMSO for 24 h and then subjected to immunoblotting analysis using antibody against p14/ARF with Actin as a loading control. **C** Downregulation of RBX1 delays the degradation and extends the half-life of p14/ARF. HepG2 and Huh7 cells were transfected with Ctrl or RBX1 siRNA for 96 h and then treated with 50 μg/mL CHX for indicated time before subjected to immunoblotting analysis. **D** MG-132 blocks p14/ARF turnover in HepG2 and Huh7 cells. HepG2  and Huh7 cells were treated with 10 μmol/L MG-132 versus DMSO in combination with 50 μg/mL CHX for indicated time, then subjected to immunoblotting using antibody against p14/ARF with Actin as a loading control. The expression of p14/ARF was quantified by densitometric analysis using ImageJ software. All data were representative of three independent experiments. Data represented means, and error bars were standard deviation.

Since RBX1 serves as an essential component of CRL E3 Ligases, we hypothesized that RBX1 regulated the degradation of p14/ARF through ubiquitin proteasome system. To test this hypothesis, small interfering RNA (siRNA) was firstly applied to knockdown the expression of *RBX1* to disassemble the CRL complex, and p14/ARF protein level was analyzed by immunoblotting subsequently in several human cancer cell lines. As shown in Fig. 2A, p14/ARF was significantly accumulated upon *RBX1* downregulation in all tested cancer cell lines. MLN4924, a small molecule inhibitor of NEDD8 activating enzyme (NAE), was applied to block Cullin neddylation and consequently inactive CRL E3 ligase activity. As shown in Fig. 2B, inactivation of CRL by MLN4924 also induced the accumulation of p14/ARF in multiple cell lines we examined. Next, the effect of *RBX1* downregulation on the degradation of p14/ARF was determined under the condition of blocking protein translation by CHX. As shown in Fig. 2C, downregulation of *RBX1* significantly delayed the p14/ARF turnover and extended the protein half-life of p14/ARF. Moreover, treatment of cells with MG-132, a classical proteasome inhibitor, also dramatically extended the protein half-life of p14/ARF compared with control cells (Fig. 2D). These findings indicated that RBX1 promoted p14/ARF degradation through the ubiquitin-proteasome system in human cancer cells.

### Cullin2 interacts with p14/ARF and mediates its degradation

**Fig. 3:**
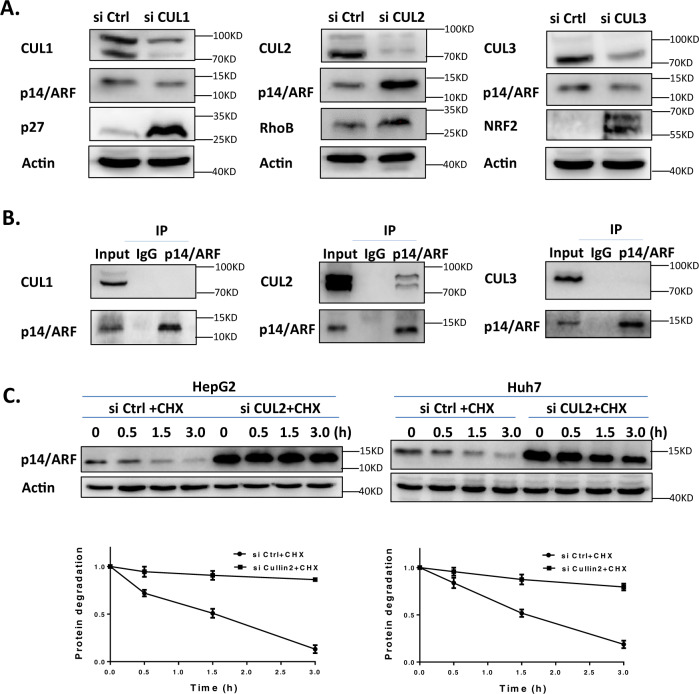
Cullin 2 interacts with p14/ARF and mediates its degradation. **A** Downregulation of Cullin 2 induces the accumulation of p14/ARF. HepG2 cells were transfected with Ctrl or Cullins siRNA for 96 h and harvested for immunoblotting using antibodies against p14/ARF and different Cullins with Actin as a loading control. **B** p14/ARF interacts with Cullin 2 at endogenous level. HepG2 cells were pretreated with MG-132 for 2 h. Cells were harvested and subjected to immunoprecipitation with anti-p14/ARF Ab and immunoblotting with anti-Cullin Abs. **C** Downregulation of Cullin2 delays the degradation and extends the half-life of p14/ARF. HepG2 and Huh7 cells were transfected with Ctrl or Cullin2 siRNA for 96 h and then treated with 50 μg/mL CHX at indicated time before subjected to immunoblotting analysis using antibody against p14/ARF with Actin as a loading control. The expression of p14/ARF was quantified by densitometric analysis using ImageJ software. All data were representative of three independent experiments. Data represented means, and error bars were standard deviation.

As RBX1 interacts with nearly all Cullin family members except Cullin5 to assemble different CRL complexes, we next determined which Cullin protein was responsible for the degradation of p14/ARF in cells. A small siRNA library targeting Cullin proteins was applied to downregulate the expression of Cullin proteins respectively, followed by the analysis of corresponding p14/ARF protein levels. We found that downregulation of *Cullin2*, but not other Cullin family members, induced marked accumulation of p14/ARF (Fig. 3A, and supplementary Fig. S1A). Moreover, immunoprecipitation with anti-p14/ARF Ab revealed the interaction between p14/ARF and Cullin2 instead of other Cullin family members (Fig. 3B, and Supplementary Fig. S1B). Finally, CHX chase assay was performed to determine the role of Cullin2 in the turnover of p14/ARF. As shown in Fig. 3C, downregulation of *Cullin2* by siRNA blocked p14/ARF degradation. These findings coherently supported the notion that CRL2 stood out of other CRL candidates to function as a novel E3 ligase responsible for p14/ARF degradation in cells.

### The CRL2 adaptor protein EloB targets p14/ARF for ubiquitination and degradation

**Fig. 4:**
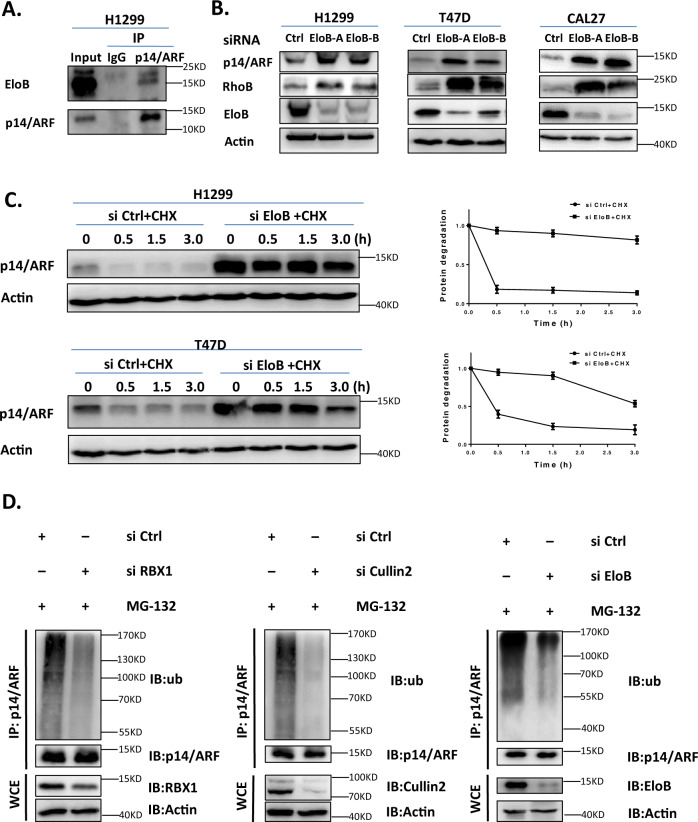
CRL2 adaptor protein EloB interacts with and mediates the degradation of p14/ARF. **A** p14/ARF interacts with EloB at endogenous levels. H1299 cells were pretreated with MG-132 for 2 h, then harvested and subjected to immunoprecipitation with anti-p14/ARF Ab and immunoblotting with EloB Ab. **B** Downregulation of EloB induces the accumulation of p14/ARF. H1299, T47D and CAL27 cells were transfected with Ctrl or Cullins siRNA for 96 h and harvested for immunoblotting using antibodies against p14/ARF and EloB with Actin as a loading control. **C** Downregulation of EloB delays the degradation and extended the half-life of p14/ARF. H1299 and T47D cells were transfected with Ctrl or EloB siRNA for 96 h and then treated with 50 μg/mL CHX for indicated time before subjected to immunoblotting analysis using antibody against p14/ARF with Actin as a loading control. The expression of p14/ARF was quantified by densitometric analysis using ImageJ software. **D** RBX1/Cullin2/EloB complex mediates polyubiquitination of p14/ARF in H1299 cells. Cells were transfected with Ctrl or RBX1, Cullin2, EloB siRNA respectively for 96 h, then treated with MG-132 for 2 h. Cells were extracted and subjected to immunoprecipitation with p14/ARF Ab and immunoblotting with anti-ub Ab. All data were representative of three independent experiments. Data represented means, and error bars were standard deviation.

After determining Cullin2-based CRL2 as a novel p14/ARF E3 ligase, immunoprecipitation with anti-p14/ARF Ab followed by mass spectrometry analysis was applied to identify the adaptor protein and substrate receptor protein responsible for p14/ARF degradation. Among those potential p14/ARF-interacting proteins, EloB, adaptor protein of CRL2, was identified as an eligible candidate (data not shown). Immunoprecipitation with anti-p14/ARF Ab and immunoblotting with anti-EloB Ab further validated the interaction between p14/ARF and EloB at endogenous levels in cells (Fig. 4A). Moreover, downregulation of *EloB* expression induced the accumulation of p14/ARF (Fig. 4B) and delayed the turnover of p14/ARF in several human cancer cell lines we examined (Fig. 4C).

As CRL2 regulating substrates degradation through UPS, the impact of RBX1/Cullin2/EloB complex inactivation on the ubiquitination of p14/ARF was further to be determined. As shown in Fig. 4D, inactivation of CRL2 with downregulation of *RBX1*, *Cullin2* or *EloB* via siRNA silencing respectively inhibited the p14/ARF polyubiquitination in cells. These findings coherently demonstrated that the RBX1-Cullin2-EloB E3 ligase complex interacted with and targeted p14/ARF for ubiquitination and degradation in human cancer cells.

### Receptor protein Prame mediates p14/ARF degradation

**Fig. 5:**
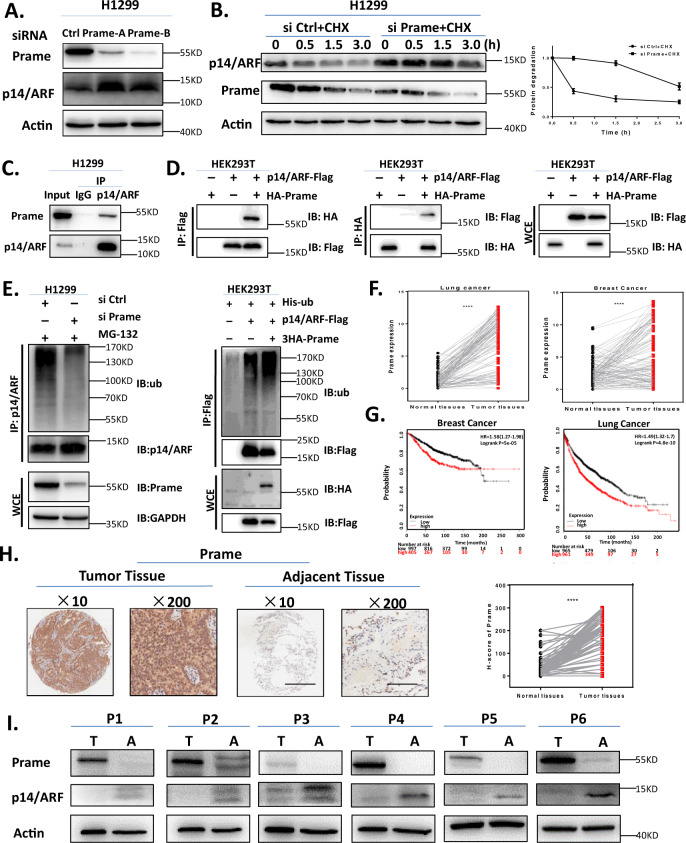
Substrate receptor protein Prame is over-expressed in human cancer and mediates p14/ARF degradation. **A** Downregulation of Prame induces the accumulation of p14/ARF. H1299 cells were transfected with Ctrl or Prame siRNA for 96 h and harvested for immunoblotting using antibodies against p14/ARF and Prame with Actin as a loading control. **B** Downregulation of Prame delays the degradation and extends the half-life of p14/ARF. H1299 cells were transfected with Ctrl or Prame siRNA for 96 h and then treated with 50 μg/mL CHX at indicated time before subjected to immunoblotting analysis using antybody against p14/ARF with Actin as a loading control. **C** P14/ARF interacts with Prame. Endogenous p14/ARF interacted with Prame. H1299 cells were pretreated with MG-132 for 2 h before harvesting and the lysates were subjected to immunoprecipitation with anti-p14/ARF Ab followed by immunoblotting with Prame Ab. **D** Exogenous p14/ARF interacted with Prame. Flag-tagged p14/ARF and HA-tagged Prame were constructed and transfected into HEK293T cells as indicated for 24 h. Total cell lysates were subjected to immunoprecipitation with anti-HA beads and immunoblotting with Flag Ab or subjected to immunoprecipitation with anti-Flag M2 beads and immunoblotting with HA Ab. **E** Prame mediates p14/ARF ubiquitination. Prame depletion suppressed ubiquitination of p14/ARF (left). H1299 cells were transfected with Ctrl or Prame siRNA for 96 h and followed with MG-132 treatment. Total cell lysates were harvested and subjected to immunoprecipitation with anti-p14/ARF Ab and immunoblotting with ub Ab. Prame overexpression promoted p14/ARF ubiquitination. HEK293T cells were transfected with indicated plasmids combinations of His-ub, Flag-p14/ARF and HA-Prame. Total cell lysates were subjected to immunoprecipitation assay with anti-Flag M2 beads and immunoblotting with ub Ab. **F**–**H** Prame is overexpressed in tumor tissues vs. adjacent tissues and indicates an unfavorable prognosis. (**F)** TCGA RNA-Seq database demonstrated Prame expression levels in breast cancer (left, *n* = 112) and lung cancer (right, *n* = 107). (**G)** Kaplan–Meier plots of OS and Prame expression in breast cancer (left, *n* = 1402, *P* < 0.0001) and lung cancer (right, *n* = 1926, *P* < 0.0001) using the program Kaplan–Meier Plotter. (**H)** Immunohistochemical staining of human lung adenocarcinoma tissue arrays using specific antibodies for Prame (*n* = 86, *P* < 0.0001). Scale bar for ×10 images: 500 μm; Scale bar for ×200 images: 25 μm. (**I)** Inverse relationship between Prame and p14/ARF. 10 paired breast cancer tissues with adjacent tissues and 4 paired lung cancer tissues with adjacent tissues were lysed and subjected to immunoblotting, the inverse relationship between Prame and p14/ARF was detected in 4/6 of paired breast cancer tissues (P1–P4, 66%) and 2/3 of lung cancer tissues (P5, P6, 66%). T: Tumor tissue, A: Adjacent tissue. All data were representative of three independent experiments. Data represented means, and error bars were standard deviation. Two‐sided *t* test.

CRL2 is a complex of Cullin2, RBX1, adaptor protein EloB/C and substrate recognizing receptor protein. The results presented above indicated that RBX1/Cullin2/EloB complex targets p14/ARF for ubiquitination and degradation. Next, our study was focused on identifying the receptor protein of p14/ARF. A small siRNA library screening targeting those potential CRL2 receptor proteins was performed, the results indicated that downregulation of *Prame* instead of other receptor proteins induced p14/ARF accumulation and delayed the degradation of p14/ARF upon CHX treatment (Fig. 5A, B, supplementary Fig. S1C). Moreover, as shown in Fig. 5C, a strong interaction between p14/ARF and Prame was detected at endogenous level in cells. Moreover, exogenous co-immunoprecipitation assay also demonstrated that Flag-p14/ARF interacted with HA-Prame (Fig. 5D). The impact of Prame on the ubiquitination of p14/ARF was further determined. As shown in Fig. 5E (left), downregulation of *Prame* via siRNA inhibited the polyubiquitination of p14/ARF. Contrariwise, up-regulation of *Prame* promoted the polyubiquitination of p14/ARF (Fig. 5E, right). These findings indicated that Prame functioned as the specific receptor protein mediating p14/ARF degradation in cells.

### Prame is up-regulated in human cancer tissues and associated with poor prognosis

To further determine the potential role of Prame-mediated p14/ARF degradation during carcinogenesis, the expression of Prame in tumor tissues and adjacent tissues were compared in the cancer genome atlas (TCGA) database. As shown in Fig. 5F, bioinformatics analysis indicated that, in lung cancer (*n* = 107) and breast cancer (*n* = 112) tissues, the mRNA levels of Prame were higher than that of the adjacent tissues. Results from Kaplan–Meier plotter, a website based on Gene Expression Omnibus (GEO) and TCGA datasets [44, 45], indicated that Prame overexpression was associated with worse overall survival (OS) [*P* = 5e − 5; HR = 1.58 95% confidence interval (CI), 1.27–1.98] in breast cancer and worse OS (*P* = 4.8e − 10; HR = 1.49; 95% CI, 1.32 − 1.7) in lung cancer (Fig. 5G) [44, 45]. Next, the expression of Prame at protein level was determined with IHC staining of tissue arrays derived from human lung cancer, which contain 86 pairs of primary tumors vs. adjacent tissues. As shown in Fig. 5H, protein levels of Prame were relatively higher in tumor tissues than that in adjacent tissues. Moreover, in Fig. 5I, the inverse relationship between Prame and p14/ARF was investigated in 10 paired breast cancer tissues with adjacent tissues and 4 paired lung cancer tissues with adjacent tissues [46–48]. As inactivation of the INK4a/ARF (or CDKN2A) locus is a common and critical genetic event in the development of human cancer, p14/ARF was deleted in four pairs of breast cancer samples and one pair of lung cancer sample we investigated (data not shown). The inverse relationship between Prame and p14/ARF was observed in 4/6 of paired breast cancer tissues (P1–P4, 66%) and 2/3 of lung cancer tissues (P5, P6, 66%) (Fig. 5I), which further confirmed the role of Prame in mediating p14/ARF degradation.

### Targeting Prame inhibits cancer cell proliferation by p14/ARF accumulation

**Fig. 6:**
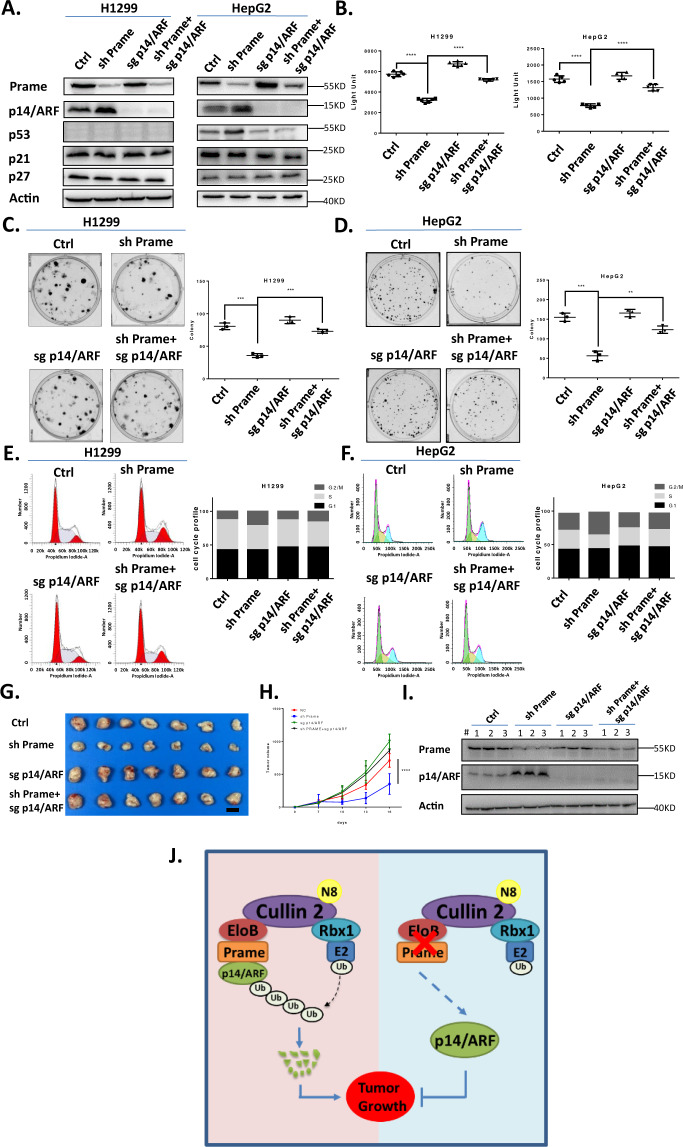
Prame serves as a novel anticancer target through p14/ARF accumulation. **A**–**D** Downregulation of Prame inhibits cell proliferation through p14/ARF accumulation in both H1299 (p53null) and HepG2 (p53WT) cell lines. Prame downregulated, p14/ARF downregulated, both Prame downregulated and p14/ARF downregulated stable H1299 and HepG2 cell lines were generated and the efficacy was demonstrated with immunoblotting (**A**), then subjected to ATPlite assay (**B**) (*n* = 5, *P* < 0.0001) and clone formation assay (**C**) (H1299, *n* = 3, *P* < 0.001), (**D**) (HepG2, *n* = 3, *P* < 0.001). **E**, **F** Downregulation of Prame expression significantly restrains cancer cell growth by inducing G2/M phase cell cycle arrest in both H1299 (p53null) and HepG2 (p53WT) cell lines. Stable cell lines mentioned above were subjected to PI staining and FACS analysis (*n* = 3, *P* < 0.0001). **G**–**I** Prame downregulated, p14/ARF downregulated, both Prame downregulated and p14/ARF downregulated stable H1299 were injected into nude mice subcutaneously (*n* = 7). (**G)** The tumor was collected immediately after the mice were euthanized. The figure shows representative tumor sizes of the 4 groups, scale bar: 10 mm. (**H)** The size of the tumors was measured with a caliper every 3 days (*n* = 7, *P* < 0.0001). (**I)** Immunoblotting of representative subcutaneous tumors derived from 4 groups stable cell lines. (**J)** Working model of this study. All data were representative of three independent experiments. Data represented means, and error bars were standard deviation. Two‐sided *t* test.

The upregulated Prame level in lung cancer implied that it may serve as an effective anticancer target. To validate this hypothesis, *Prame* was downregulated with shRNA in both H1299 (p53null) and HepG2 (p53WT) cell lines. Cell viability assay (ATPlite luminescence assay) and clonogenic assay demonstrated that cell proliferation was inhibited upon downregulation of Prame, which could be rescued by simultaneous downregulation of p14/ARF in both H1299 (p53null) and HepG2 (p53WT) cell lines (Fig. 6B–D). After determining the anticancer effect of targeting Prame, we analyzed the cell cycle status of *Prame*-downregulated H1299 and HepG2 cells lines with PI staining and FACS analysis. As shown in Fig. 6E, G2/M phase cell‐cycle arrest was observed when downregulating *Prame* expression, which could also be rescued by simultaneous knocking down of p14/ARF in both H1299 and HepG2 cell lines. Moreover, as shown in Fig. 6A, downregulating of *Prame* did not affect the expression status of other cell cycle inhibitor like p21, p27. Altogether, we concluded that Prame-p14/ARF axis regulated cell cycle progression in both p53WT (HepG2) and p53null (H1299) cells.

After determining the inhibitory effect of targeting Prame on cancer cell proliferation in vitro, we validated the effect of targeting Prame on tumor growth in vivo. We first constructed Prame downregulated, p14/ARF downregulated and both Prame and p14/ARF downregulated stable H1299 cell lines. Nude mice were randomized into four groups (*n* = 7) and injected with stable cell lines described above subcutaneously. As shown in Fig. 6G, H, compared with negative control group, the tumor formation of Prame downregulated group was inhibited. Consistently, tumor growth suppression induced by Prame downregulation was rescued by simultaneous downregulation of p14/ARF, suggesting the inhibitory effect of targeting Prame on tumor formation was mediated by p14/ARF accumulation (Fig. 6G–I).

## Discussion

When it comes to the turnover of p14/ARF, Chen et al. [49] first identified ubiquitin ligase for ARF (ULF)/TRIP12 as the E3 ubiquitin ligase mediating p14/ARF degradation in normal human cells. However, Chen et al. noted that in cancer cells ULF mediating p14/ARF degradation was abrogated due to the commonly overexpressed nucleophosmin (NPM) and c-Myc, which made the degradation of p14/ARF in cancer cells remain elusive [49, 50]. In this study, we determined that in human cancer cell lines p14/ARF was degraded by CRL2^Prame^ E3 ubiquitin ligase. Moreover, targeting overexpressed Prame in human cancer cell lines induced G2/M phase cell cycle arrest and inhibited cancer cell proliferation by blocking p14/ARF degradation (Fig. 6J).

RBX1, the RING component of CRL E3 ubiquitin ligases, regulates diverse cellular processes by targeting a variety of substrates for degradation [27, 28]. In this study, by proteomic analysis of RBX1-interacting proteins combined with in-depth and logical mechanistic studies, p14/ARF was identified as a new substrate of RBX1-Cullin2-EloB-Prame E3 ligase complex (CRL2^Prame^). CRL2 is a complex of Cullin-2, RBX1, adaptor protein EloB/C and substrate receptor protein [51]. HIF1α is the best known CRL2 substrate that binds to the substrate receptor protein VHL for ubiquitination and degradation [52, 53]. In von Hippel–Lindau (VHL) syndrome, mutated VHL breaks its interaction to adaptor protein and inactivates the CRL2 E3 ligase, which leads to the accumulation of HIF1α and promotes angiogenesis, proliferation and cell survival by transactivating many of its target genes involved in regulation of these processes [54, 55]. Besides HIF1α, oncogenic proteins β2-adrenergic receptor (β2-AR), atypical PKCλ, and the RNA polymerase II subunit hsRPB7 are also identified as the substrate of CRL2 [56–58]. Therefore, in contrast with CRL1 complex regulating the degradation of many tumor suppressors, such as p21, p27, Iκ-Bα and NF1, CRL2 seems to mainly target oncogenic substrates for degradation [24, 59]. Unlike previous study, here we reported p14/ARF as tumor-suppressive substrate of CRL2. Additionally, in our recently published study, we also identified the tumor suppressor RhoB as a substrate of CRL2 [60]. These findings unraveled a new profile of CRL2 and challenged the conventional concept of CRL2 as a potential tumor suppressive agent.

In the article published in 2004, Kuo et al. [61] reported that polyubiquitin was attached to the Nterminus of ARF. As N-terminal ubiquitination and acetylation might occur competitively [62], Kuo et al. engineered ARF mutants that were more efficiently to be acetylated at N terminus and analyzed the extent of polyubiquitination level of those ARF mutants. The result turned out that ARF mutants underwent polyubiquitination to a lesser extent and were more stable, demonstrating that polyubiquitin was conjugated to the N terminus of ARF [61]. However, the mechanism of N-terminal polyubiquitination on lysine-less human p14/ARF remains unclear. This might be explained by that some ARF protein misincorporate lysine for arginine during translation [61]. Despite the fact that ARF contains∼22% arginyl residues, the different arginyl tRNAs in mammalian cells are relatively abundant, and the misincorporation rate of lysine for arginine in cell-free reticulocyte translation systems is very low (0.06%–0.2%) [63]. Therefore, further research is required to reveal the mechanism of N-terminal polyubiquitination on lysine-less human p14/ARF and to determine the certain amino groups of p14/ARF undergo N-terminal polyubiquitination.

Altogether, in this study, we revealed the overexpressed status and the prognostic values of CRL2 receptor protein Prame in human cancer tissues, estimated the inhibitory effects of blocking CRL2 complex through targeting Prame on cancer cell proliferation. Most importantly, tumor suppressor p14/ARF was identified as the first substrate of CRL2^Prame^, which revealed a novel mechanism for Prame as an oncogenic factor and therapeutic target.

## Materials and methods

### Cell lines and cell culture

Human hepatocellular carcinoma cell line HepG2, lung cancer cell line H1299, breast cancer cell line T47D, kidney cancer cell line CAL27 were obtained from the American Type Culture Collection, Human hepatocellular carcinoma cell line Huh7 was purchased from the Cell bank of the Type Culture Collection of the Chinese Academy of Sciences (Shanghai, China) and routinely cultured as previously described [64]. All cell lines applied were recently authenticated and tested without mycoplasma contamination.

### Co-IP/MS

Co-IP/MS was performed as previously described [65]. Briefly, HepG2 were lysed using pre-chilled NP-40 lysis buffer (1 mL/e7 cells, 50 mM pH7.5 Tris-HCl, 150 mM NaCl, 0.5% NP-40, and 1 mM PMSF) on ice. 1 mL WCE (whole cell extracts, ~10 mg) was incubated with 7 μg primary antibody for 2 h followed by ultracentrifugation and 45 min incubation with 10 μL Protein A beads (GE healthcare) in 4 °C. Beads were collected by centrifugation and washed 3 times with NP-40 lysis buffer. Immunocomplexes were eluted by Laemmli buffer and separated by SDS-PAGE for 2 cm on separation gels. Gels were minimally stained with Coomassie brilliant blue and cut into 6 molecular weight ranges as well as a heavy chain IgG band. The cut gels were destained with destain buffer (40% methanol, 50 mM NH_4_HCO_3_) and digested with trypsin. Immunocomplexes were identified on a Thermo Fisher Orbitrap Fusion Lumos Mass Spectrometer. Raw data were uploaded and searched against human protein RefSeq database in Firmiana [66].

### Immunoprecipitation

To immunoprecipitate endogenous proteins, cell extracts were incubated with primary antibodies p14/ARF (AB11048, Abcam), RBX1 (ab133565, Abcam) or control IgG in a rotating incubator overnight at 4 °C, followed by incubation with protein A/G magnetic beads (Bimake) for another 2 h. The immunoprecipitates were washed three times with lysis buffer and analyzed by immunoblotting.

### Gene silencing using small interfering RNA (siRNA)

Cells were transfected with siRNA oligonucleotides synthesized by Genepharma (Shanghai, China) using Lipofectamine 2000. The sequences of the siRNA were as follows: RBX1, 5′-GACTTTCCCTGCTGTTACCTAATT-3′, 5′-CTGTGCCATCTGCAGGAACCACATT-3′; Cullin1, 5′-CUAGAUACAAGAUUAUACAUGCGG-3′; Cullin2, 5′-GCACAAUGCCCUUAUUCAA-3′, 5′-GCAGAAAGACACACCACAA-3′; Cullin3, 5′-TTGACGTGAACTGACATCCACATTC-3′, 5′-TACATATGTGTATACTTTGCGATCC-3′; Cullin4A, 5′-GAAGAUUAACACGUGCUGGTT-3′; Cullin4B, 5′-AAGCCUAAAUUACCAGAAA-3′, 5′-GGAGUUAUUUAGGGCUCAU-3′; Cullin5, 5′-CUACUGACUCUGAGAAAUA-3′, 5′-GAGCAAAUAGAGUGGCUAA-3′; Cullin7, 5′-UGAGAUCCUAGCUGAACUG-3′, 5′-UGUCCAAGGAUGAGAUCUA-3′; EloB, 5′-UGAACAAGCCGUGCAGUGA-3′, 5′-AGCGGCUGUACAAGGAUGA-3′; Prame, 5′-CCUGGAAGCUACCCACCUUTT-3′, 5′-GCUCCCAGCUUACGACCUUTT-3′; LRR1, 5′-CCUGUGGAUAUCUGUCUAATT-3′, 5′-GCUCUCAUAUCAUUCCAUUTT-3′; FEM1B, 5′-GCCCGCAAUGGACACGCAATT-3′, 5′-CCUAAUGAUUGCGGCAUAUTT-3′; VHL, 5′-GCCAGUGUAUACUCUGAAATT-3′, 5′-GCUCUACGAAGAUCUGGAATT-3′, 5′-TTGTGCCATCTCTCAATGTTGAC-3′.

### Immunoblotting and cycloheximide (CHX)-chase assay

Cell lysates were prepared and analyzed by immunoblotting. Antibody against p14/ARF (AP51072) was purchased from ABGENT (Suzhou city, Jiangsu Province, China). Antibodies against RBX1 (ab133565), Cullin1 (ab75817), Cullin2 (ab166917), Cullin5 (ab18477), Cullin7 (ab96861), EloB (ab151743), Prame (ab219650) were purchased from Abcam (Shanghai, China). Anitbodies againgst Cullin3 (2759), Cullin4A (2699) were from Cell Signaling Technology (Danvers, MA, USA). Antibody against Cullin4B (12916) was from Proteintech (Wuhan, Hubei, China).

For CHX-chase experiments, cells were treated with 50 μg/mL CHX (Sigma, C4859, Merck KGaA, Darmstadt, Hesse, Germany) in combination with 1.0 μmol/L MLN4924 or DMSO for indicated time points [64].

### In vivo ubiquitination assay

To detect endogenous p14/ARF ubiquitination, H1299 cells were transfected with siRNA oligonucleotide targeting RBX1, Cullin2, EloB, Prame along with scrambled control siRNA. At 96 h post-transfection, cells were treated with MG-132 for 2 h. Then cells were lysed with 100 μL cell lysis buffer (Thermo Fisher) with 1% SDS per plate. Cell lysates were boiled for 10 mins and then diluted in 10 volumes of lysis buffer without SDS. Then samples were subjected to immunoprecipitation with anti-p14/ARF Ab (ab3642) from Abcam (Shanghai, China) and immunoblotting with anti-ubiquitin Ab (sc8017) from Santa Cruz Biotechnology (Dallas, Texas, USA).

### Collection of tumor tissues and ethics statement

For immunohistochemically (IHC) assay, fresh primary lung cancer tissues and their adjacent lung tissues were collected from 86 lung adenocarcinoma patients undergoing resection from July 2004 to June 2009, at the Taizhou Hospital (Taizhou). For immunoblotting assay, 4 pairs of adjacent lung tissues and tumorous lung tissues of patients were collected in Xinhua Hospital of Shanghai Jiaotong University School of Medicine (Shanghai). 10 pairs of adjacent breast tissues and tumorous breast tissues of patients were collected in Fudan University Shanghai Cancer Center (Shanghai). Written informed consent regarding tissue and data use for scientific purposes was obtained from all participating patients. The study was approved by the Research Ethics Committee of Taizhou Hospital, Xinhua Hospital and Fudan University Shanghai Cancer Center in agreement with the Declaration of Helsinki.

### Cell viability assay and clonogenic survival assay

Cells were seeded in 96-well plates (3 × 10^3^ cells/well), cell proliferation was determined using the ATPLite Luminescence Assay kit (PerkinElmer, Waltham, MA, USA) according to the manufacturer’s protocol. Five independent experiments were performed. For the clonogenic assay, 200 H1299 cells and 300 HepG2 cells were seeded in 6-well plates and incubated for 10 days. Colonies comprising 50 cells or more were counted under an inverted microscope [64]. Three independent experiments were performed.

### Bioinformatics analysis

TCGA RNA-Seq and corresponding clinical data were based upon data generated by TCGA Research Network (https://cancergenome.nih.gov/). RNA-Seq analysis was performed for the data from 112 pairs of breast cancer tissues with adjacent tissues, and 107 pairs of lung cancer tissues with adjacent tissues.

### Immunohistochemical staining of human tumor tissue array

Human lung cancer tissue arrays were IHC stained with Prame antibody from Shanghai Biochip (Shanghai, China). The tissue array sections (5 microns) were dehydrated and subjected to peroxidase blocking. Primary antibody was added and incubated at room temperature for 30 min on the DAKO AutoStainer using the DakoCytomation EnVision+ System-HRP detection kit (Dakocytomation, Carpinteria, CA). The slides were counterstained with hematoxylin. The stained slides were observed under microscopy, and images were acquired. Immunohistochemistry results was further evaluated by a semiquantitative approach H-score (or “histo” score) ranging from 0 to 300. For each sample, H-scoring assessment was estimated by multiplying staining intensity (0, negative; 1+, weak; 2+, moderate; and 3+, strong) together with the percentage of positive cells (0–100%) using formula: [1 × (% cells 1+) + 2 × (% cells 2+) + 3 × (% cells 3+)] [67, 68]. Interpretation of IHC results were performed by two independent scientists. Samples were excluded if they were unpaired.

### Generation of stable cell lines

Prame knockdown was performed with GIPZ lentiviral short hairpin RNA (shRNA) vectors expressing Prame shRNA (shPrame: oligos 5′-CCUGGAAGCUACCCACCUUTT-3′) obtained from GENECHEM (Shanghai, China). P14/ARF knockdown was performed by p14/ARF sgRNA oligos: forward 5′-CACCGTCTTGGTGACCCTCCGGATT-3′ and reverse 5′-AAACAATCCGGAGGGTCACCAAGAC-3′ into LentiCRISPR v2 plasmid (Plasmid #52961, Addgene, Cambridge, MA, USA). LentiCRISPR plasmid with SgRNA (4.0 μg, packaging plasmids psPAX2 (3.0 μg) and pMD2.G (1.0 μg) were transfected into HEK293T cells with Lipofectamine 2000, and virus supernatant was harvested 36 h post transfection and mixed with polybrene to increase infection efficiency. The infected H1299/HepG2 cells were selected with fluorescence activated cell sorting (FACS) or 2 μg/mL puromycin for 2 weeks [69].

### Subcutaneous xenograft in mice

Female BALB/c athymic nude mice (ages 4–6 weeks) were randomized into four groups and treated in accordance with established guidelines, and the protocol was approved by an internal animal protocol review committee. Tumor xenografts were measured with a caliper every 3 days, and tumor volume was determined using the formula: (length × width^2^)/2. The investigators were blinded to the group allocation during the experiment and when assessing the outcome. Subcutaneous tumors were collected and subjected to immunoblotting analysis after mice were sacrificed.

### Statistical analysis

The statistical significance of differences between groups was assessed using GraphPad Prism6 software. The student *t* test was used for the comparison of parameters between groups. Data are presented as mean ± standard deviation. For all tests, four levels of significance (*P < 0.05, **P < 0.01, ***P < 0.001, ****P < 0.0001) were applied [64].

## Supplementary information

SUPPLEMENTAL Figure 1
