# Supplementary figures and images for: Tumor-associated antigen Prame targets tumor suppressor p14/ARF for degradation as the receptor protein of CRL2Prame complex
Source: Cell Death Differ. 2021 Jan 27;28(6):1926–40. doi: 10.1038/s41418-020-00724-5 (PMC8184998; doi:10.1038/s41418-020-00724-5)

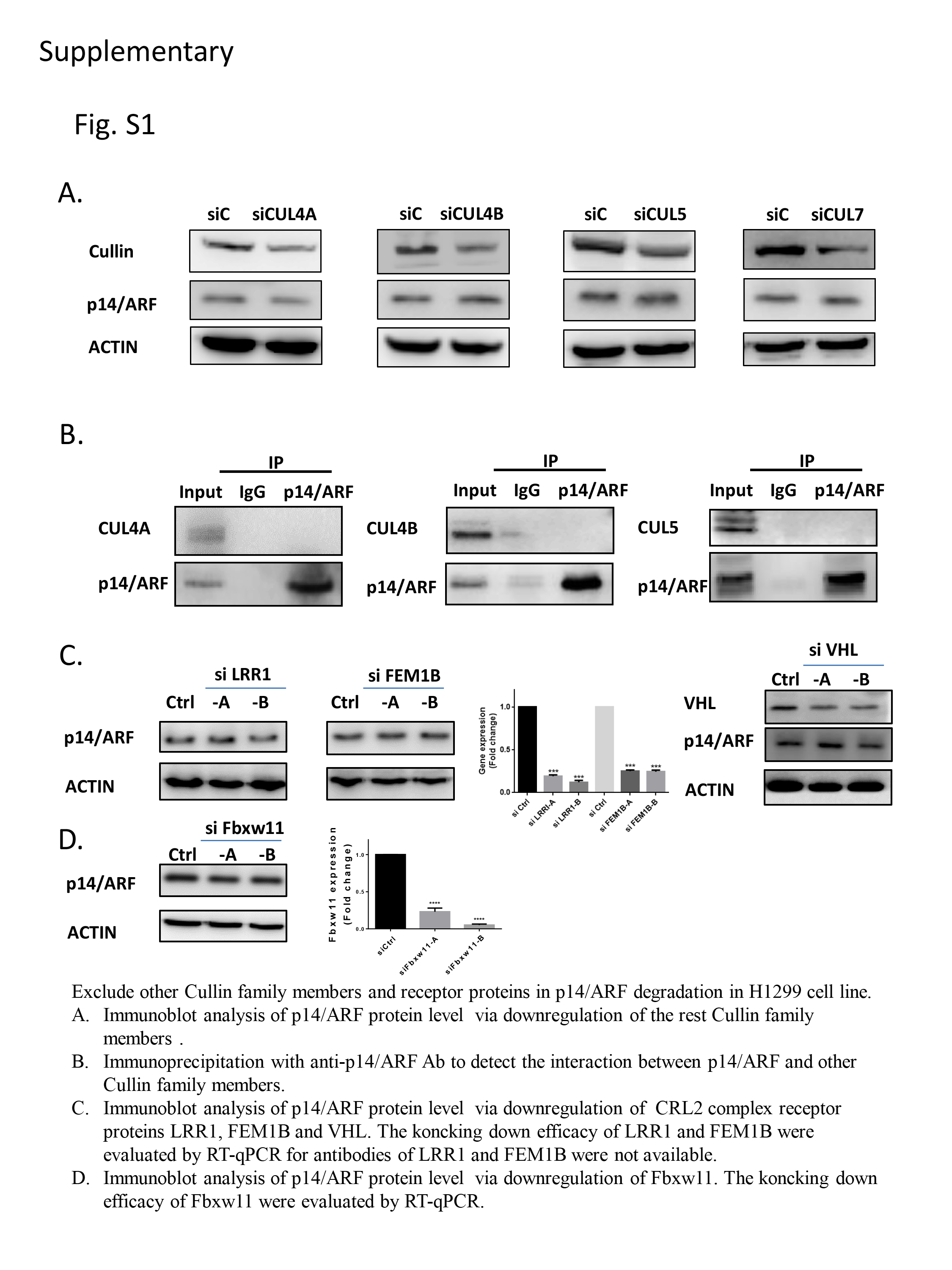

Supplement: Supplementary file 1 — SUPPLEMENTAL Figure 1 [file 41418_2020_724_MOESM1_ESM.png]
